# Gender roles perceptions and ideal number of children: case study of Emirati youth

**DOI:** 10.1186/s12978-023-01677-x

**Published:** 2023-09-13

**Authors:** Ankita Shukla, Tatiana Karabchuk, Latifa Mohammed Al Neyadi

**Affiliations:** 1 Research Institute of Medical and Health Sciences, University of Sharjah, Sharjah, United Arab Emirates; 2 Department of Government and Society, College of Humanities and Social Science, United Arab Emirates University, Al Ain, United Arab Emirates; 3 National Research University Higher School of Economics (Associate Researcher), Moscow, Russia; 4 UAEU Library, United Arab Emirates University, Al Ain, United Arab Emirates

**Keywords:** Gender roles, Ideal number of children, Gender equality attitudes, Youth, Fertility

## Abstract

**Background:**

The United Arab Emirates (UAE) is a traditional society with patriarchal values. The country has been experiencing a decline in fertility rates, bringing the total fertility rate for the national population to 3.3 children per woman, the lowest since 1970s. Existing literature indicates that having gender-egalitarian attitudes is associated with lower fertility rates. Therefore, this study aims to investigate the perceptions of gender roles among the highly educated Emirati youth and examine whether these attitudes influence their desire to have children. By doing so, we aim to gain insights into the factors contributing to the country’s declining fertility rates.

**Methods:**

This study utilized data from a cross-sectional quantitative survey. The survey was developed and administered in February–March 2019 to a purposive sample of 300 young Emirati males and females aged 18–30 years, studying at the UAE University. Both bivariate and multivariate analysis were performed to examine the levels of youths’ perception towards gender roles and desired fertility.

**Results:**

The data collected from Emirati youths revealed that 50% of them supported the traditional perspective on marriage, 30% considered motherhood is the most important thing for women, and a small percentage supported economic independence of women and husband participation in household chores/child-rearing. On average Emirati youth ideally wished to have 5.4 children in their future families, with a preference for sons over daughters. Youths who favoured women’s economic independence and equal participation in household work contribution by spouses desired a lower number of children which is in line with the modernization theory and cultural evolution.

**Conclusion:**

The UAE has been facing continuous decline in fertility rates. The study indicates that ideal number of children is much higher than the current fertility rates in the country. To bridge this gap, the government could implement family policies that create an environment conducive to fulfilling the ideal desires of young Emiratis regarding family size. Moreover, present findings indicate that perceptions of equal gender roles could be a contributing factor to the declining fertility rates among the young generation. Promoting gender equality attitudes and strengthening institutional support for childbearing could become key strategy to address these issues.

## Introduction

The UAE has been considered to be a culturally conservative society, characterized by tribal affiliations and a patriarchal family structure [1]. Previous studies have shown that Emirati women are often discouraged from working outside the home due to cultural barriers in a patriarchal Islamic society, that views women primarily as housewives and caregivers [2]. However, over the past two decades, there have been significant changes in people’s attitude towards everyday life in the country.

The UAE government’s female supportive policies have widened education and employment opportunities for women [3]. In fact, since 2010 higher education has been dominated by women in the country [4]. Moreover, the female labour force participation rate has almost doubled since 1990s and reached 57% in 2019 [5]. This is comparable to the female labour force participation rates in United States (57%), Germany (57%), Austria (56%), Luxembourg (56%), France (51%), and South Korea (54%) [5]. All these changes compounded with the rapid expansion of information technology, are assumed to have influenced attitudes towards women, gender roles and relationships between women and men in society. This raises the question of whether these rapid cultural and social changes have any influence on the fertility patterns among Emirati youth.

A significant shift has taken place in the fertility rates of the Gulf Cooperation Council (GCC) countries since 1990. Between 1990 and 2009 all GCC countries experienced a dramatic decline in their fertility rates, including the UAE. The total fertility rate (TFR) in the UAE has declined from 4.5 children per woman in 1990 to 1.4 in 2019 [6]. When analysing these fertility rates, it is very important to keep in mind what the country has high proportion of expatriates in its population. These expatriates are primarily here for work and do not have as many children as Emirati citizens. Moreover, the UAE population was much smaller in 1990, and the proportion of expatriates was not as big as in present. In 2019 the percentage of expatriate population reached 88%. Thus, the dynamics of the fertility rates among Emirati women differ considerably. The fertility rates for the national population has dropped from 6.7 children per woman in 1970 to 3.7 by 2017 [7].

The ideal or desired number of children is often considered as a proxy measure of fertility specially for individuals who have not yet completed their fertility. Previous studies have shown that fertility intentions are reliable predictors of future fertility [8–10]. The desired number of children among the younger generation is often influenced by societal norms and/or their family background. Evidence from Europe suggests that areas with shared cultural characteristics have experienced a comparable decline in fertility, irrespective of their level of development [11].

Women from religious family backgrounds, large families, and households where adults prefer a larger family size tend to have higher desired number of children [12, 13]. Social attitudes, beliefs, and values are identified as significant factors influencing desired fertility. Individuals with stronger religious beliefs and traditional gender values, tend to have increased likelihood for higher number of desired children or larger families [14, 15]. Conversely, educated and working women, tend to desire a smaller number of children [16–18].

Plenty of research is available linking fertility behaviour and attitudes towards social gender roles in developed countries. However, there is not much information available on the Gulf region. Present study fills the gap by investigating the relationship between youth attitudes towards equitable gender roles and their desired number of children. The study additionally examines the effect of mother’s fertility and time spent by parents in household activities on youth’s ideal number of children. Such findings are needed to inform future policies that can help to balance country’s population structure and encourage youths to have more children.

## Country background

The United Nation (UN) estimated the country’s total population to be 9,400,145 as of mid-year 2017, with immigrants making up more than 88% of the total population. The UAE has the 7th highest net migration rate in the world at 12.36 [19]. Consequently, the country’s population structure is exceptionally diverse, representing more than 200 nationalities within its borders.

The TFR for Emirati women (aged 15–49 years) has declined from 6.7 births per woman in 1970s to 3.7 in 2017 [20]. Similar declines in fertility rates have been witnessed in all GCC Countries. For instance, Oman had 7.2 births per woman in 1960 which dropped to 2.8 in 2019 [21].

The declining birth rates in the Gulf are attributed to a number of factors, including urbanization, delayed marriage, changing attitudes to the family size, and increased education and work opportunities for women [1, 3, 22]. A review of the existing literature reveals a scarcity of empirical data on this issue. Only a few publications have tackled the dynamics of fertility rates in the UAE and it’s rare to find research on desired fertility or ideal number of children.

Al Awad and Chartouni (2014) examined the factors that contributed to the decline in fertility in the GCC countries in recent years, taking the UAE as a case study. They found that economic factors, such as the cost of raising children in the UAE, are not essential determinants of fertility due to the large amount of social insurance provided by the UAE Government. Furthermore, labour market participation by either males or females does not play a critical role in determining fertility in the UAE. The two primary causes of decline in fertility rates are late marriages or late first births and higher levels of female education. Other contributors to the fall in fertility rates are marriages between UAE national males and foreign females and an increase in birth intervals. Inversely, the size of household residences and the number of domestic workers working in households contribute positively to fertility. To examine the effects of education, employment, and income on the reproductive attitudes and behaviours of married women in the UAE, Alibeli (2014) conducted a study and interviewed 1030 married women from the country’s seven emirates. The study found that there were no significant effects of education, employment, and income on the reproductive attitudes and behaviours of the respondents. Previous studies have focused on demographic (age of first marriage, mixed marriages) and economic (education, employment, income) factors of fertility.

Data from cross-national surveys in the Arab region show that support for equal gender roles is generally increasing among the youngest generations, even in the conservative settings [23]. Belief in equal gender roles or equalitarian society has already been linked with lower fertility intentions in the literature [15]. Indeed, the UAE is doing very well in terms of female empowerment and gender equality, ranking 18th globally on Gender inequality index (GII).^1^ This makes the UAE the most gender equal country in the Arab region [24]. For a country like the UAE, which is already dealing with a declining fertility rate and an increasing inclination towards equal gender attitudes, it is essential to investigate the links between gender attitudes and desired fertility. Hence, there is a need to examine societal norms and expectations, gender roles, gender equality attitudes in relation to the ideal number of children.

## Literature review

The ideal number of children, or desired fertility, reflects the fertility preferences that are often defined by societal fertility norms. People develop their ideas about the preferred number of children during their socialization and through their life experiences. Since 1936, the concept of the “ideal number of children” has been used to measure attitudes toward fertility and particularly toward population growth [25]. The ideal (or desired) number of children has been proven to be highly predictive of subsequent fertility [8, 9]. Fertility desires lay the foundation of fertility intentions, which in turn, transform into real children [26]. Indeed, according to the Theory of Planned Behaviour, individuals make reasoned and logical decisions to engage in a particular behaviour by considering the available information [27]. The behaviour performance is determined by the individual’s intention to engage in it and the perceived control insight they have over the behaviour [27].

Research from Western societies has revealed that social, economic, and cultural changes have influenced women’s employment, marriage patterns, and the decrease in fertility rates [28–30]. It is argued that an interplay between systemic-level explanations such as improved fertility control measures, changes in the labour market, and changing gender roles, and individual level explanations such as individualization, risk and the social values of late modernity are the causes of fertility decline [31, 32]. Population economic theories have linked low fertility with increasing female economic independence [33] and the opportunity costs of childbearing for women [34, 35].

However, it is also argued that women’s fertility behaviour and preference are not only influenced by their participation in paid work, but also by the burden of work on them in their homes [36]. The distribution of housework responsibilities in the domestic sphere has been rather unequal between men and women, especially in the Gulf. Gender role attitudes reflect the distribution of professional and housework responsibilities between women and men [37]. Often, the gender roles assigned to men and women in society define fertility behaviour [38, 39]. It is indicated that women and couples with egalitarian attitudes are less likely to intend for an additional child compared to women and couples with traditional mindsets [15, 40]. It should be kept in mind that the relationship between gender role attitudes and fertility intentions varies depending on the measurement of gender role attitudes, the gender of the study subjects, parity and social contexts [10, 41].

Kaufman (2000) found that the difference between men and women in their gender role attitudes significantly affects desired fertility. Egalitarian women expect support from their partner in household chores and child rearing, and in absence of this support they tend to have/desire fewer children. However, egalitarian men who want to be part of the child rearing process desire more children [15]. Examination of the slight reversal of fertility in the developed countries has revealed that changing roles of men in the home and family are one of the contributors to the increase in fertility [42]. McDonald (2000b) also shares similar view, examining the difference in gender equity in institutional and social context and the shift towards lower fertility [43].

The second demographic transition was slow in the Middle East due to strong cultural norms and traditions [44]. However, recently urbanization, industrialization and modernization have led to changes in lifestyle, development and education [45]. Female empowerment has influenced the ideal number of children by increasing the demand for smaller families [46]. A study from Saudi Arabia demonstrated that education has a high extrapolative effect on the age of marriage, the age of husbands at marriage, the use of contraception and perceptions of the ideal size of a family [47].

It has already been shown in the literature that employment, income, and education of women do not have a significant effect on reproductive behaviour and attitudes in the UAE [1]. Since the UAE is a traditional society with high respect for social norms, we assume that attitudes towards gender roles and gender equality would explain the variance in the desired number of children. In this paper, we assessed two hypotheses. Hypotheses 1: whether there is any association between gender equality attitudes and ideal number of children Hypotheses 2: whether there is any association between number of siblings in the parental family and housework distribution between father and mother and preferred ideal number of children among Emirati youths.

## Methodology

### Data description

This study utilized data from a cross-sectional quantitative paper survey conducted in February–March 2019. The survey was administered to a purposive sample of undergraduate Emirati male and female students, aged 18–30 years, who were studying at the UAE University. Data was collected via face-to-face interviews using a structured questionnaire on family background, demography, economic activities, gender value measures and desired fertility. The survey data file could be sent at any time by email upon request to the corresponding author.

The interviews were conducted in Arabic language by trained licensed interviewers with prior expertise in conducting structured face-to-face interviews. The questions were read by the interviewers and the answers were written down accordingly. Before the start of any interview the interviewers were briefing about the goal of the study and participants rights to withdraw any minute. All the participation in the study was on the voluntary basis, confirmed by written consent form. No personal information was asked or recorded; all answers were kept in complete anonymity. Each interview lasted between 15 and 30 min. The analysis was restricted to 300 respondents who provided their answers for all the questions on gender equality attitudes and desired number of children.

### Ethics considerations

The UAE University’s Social Research Ethical Committee approved the study protocol and materials before data collection. Participation in this study was voluntary and each participant was asked to sign the written informed consent form before taking part in the survey. To safeguard participants’ privacy, confidentiality, and anonymity, no personal information was recorded or asked; measures were taken to de-identify the data collected during the study via random coding. Each participant was explicitly informed of their right to withdraw from the study at any juncture without the need for an explanation, and if they wished their collected data will be permanently deleted.

### Variables

Dependent variable for this study was the ideal number of children, measured through the question: “What is the ideal number of sons and daughters for you to have in your family”? The number of sons and daughters were summed to generate the variable “ideal number of children”.

Independent variables: main predictors of this study included statements on gender equality and perceptions of gender roles. The scales/statements were borrowed and adopted from the cross-national surveys such as International Social Program (ISSP), General Social Survey (GSS), World Value Survey (WVS) and European Value Study (WVS) questionnaires, Gallup World Poll, PEW National Parental Survey and OECD (see detailed description with the reference to the sources in the annex Table 5). The borrowed statements on gender equality and distribution of the roles between men and women were validated and tested scales by the originators of the surveys. The authors added two new items on traditional norms and equal loads of the housework. The statements/items were grouped into the following five domains on gender equality attitudes: This categorization was determined by the authors based on the similarity or likeness among the statements.

I. Negative impact of working mother on child-rearing
  1. A working mother can establish just as warm and secure a relationship with her children as a mother who does not work (reverse coded)
  2. A pre-school child is likely to suffer if his or her mother works

II. Importance of motherhood and family for women
  1. A job is alright but what most women really want is a home and children
  2. Being a housewife is just as fulfilling as working for pay

III. Husband/male equal participation in household (HH) work and child-rearing
  1. Both husband and wife should equally do the work on household (cooking, cleaning, shopping, repairing etc.)
  2. Men should take as much responsibility as women for the home and children
  3. In general, fathers are as well suited to look after their children as mothers

IV. Importance to follow the traditions
  1. When deciding about the marriage partner the most important is to follow the advice of parents

XXII. Importance of economic independence of women and gender equality at the labour market
  1. Having a job is the best way for a woman to be an independent person
  2. When jobs are scarce, men have more right to a job than women (reverse coded)
  3. It is better for a family if husband earns more than wife (reverse coded)

Respondents were asked to indicate the extent to which they agree or disagree with these statements on a scale of 1–4 where 1 is strongly agree, 2 is agree, 3 is disagree and 4 is strongly disagree. All statements were re-coded in two categories 0-Disagree and 1-Agree. A score was created by summing responses of all statements under each domain. Here higher scores indicate strong agreement with the statements.

The distribution of the sum of all statements was examined and cut-off points were created at the 50th percentile (median). Further they were categorized into two levels– agree and disagree. Scores lower than 50% of the distribution were categorized as ‘disagree’ meaning respondent disagreed with the given statements; and scores above 50% were categorized as ‘agree’ meaning respondents agreed with the given statements.

As per the literature gender [48], age [49], marital status, paternal education [50], household wealth [49], and maternal reproductive behaviour [10, 51] have been found to be potential determinants of the ideal number of children. The present study also analysed these factors as independent variables– gender (male: female), age (19–22:23–30), marital status (unmarried: married), father’s education (Primary or less: Basic secondary: High school: Undergraduate: Post graduate and above) mother’s education(Primary or less: Basic secondary: High school: Undergraduate: Post graduate and above), average hours per week spent by father in HH work, average hours per week spent by mother in HH work, status in social ladder (0:10) and number of children born to mother (1:19).

### Statistical analysis

Both bivariate and multivariate analysis^2^ were performed to examine the levels of youth’s attitude towards gender equality attitudes and desired fertility. Ideal number of children is calculated as mean. Bivariate analysis included chi-square test to test the association between background characteristics and gender equality attitudes, and t-test to test the difference in mean ideal number of children by gender equality attitudes. Multivariate analyses included Poisson regression estimation to test the hypotheses about the relationship between gender equality attitudes and desired number of children. Poisson regression is often used for modelling count data [52]. It assumes the response variable ‘Y’ has a Poisson distribution and assumes the logarithm of its expected value can be modeled by a linear combination of unknown parameters. In this analysis, Y is “ideal number of children by the participants”, and ranges from 0 to 16, with a mean of 5.4. To assess the fit of the model, we performed the goodness-of-fit chi-squared test. The goodness-of-fit chi-squared test was not statistically significant, which indicated that Poisson model was appropriate.

## Results

Forty-two percent of participants in the sample were males and 58% were females (Table 1). Age of the participants ranged between 19 and 30, with 59% in 19–22 age group and 41% in the 23–30 age group. Majority of the sample was unmarried (91%) with only 9% being married at the time of the survey (due to the sample bias towards not married respondents this variable was excluded from the regression analysis). About 16% of participants’ mothers and fathers had primary or less education, and only 7% had fathers and 5% had mothers educated at university and above levels.

**Table 1:** Sample distribution of Emirati youths, 2019

	N	Percent
Gender		
Male	126	42.0
Female	174	58.0
Age		
19–22	178	59.3
23–30	122	40.7
Marital status		
Unmarried	274	91.3
Married	26	8.7
Father’s education		
Primary or less	49	16.5
Basic secondary	48	16.2
High School	94	31.7
Undergraduate	86	29.0
Graduate and above	20	6.7
Mother’s education		
Primary or less	46	15.8
Basic secondary	46	15.8
High School	109	37.3
Undergraduate	76	26.0
Graduate and above	15	5.1
Number of children born to mother		7.1 (mean)
Hours spend in household activities by father		15.8 (mean)
Hours spend in household activities by mother		24.5 (mean)
Social ladder (range: 1–10)		6.6 (mean)
Total	300	

**Fig. 1:**
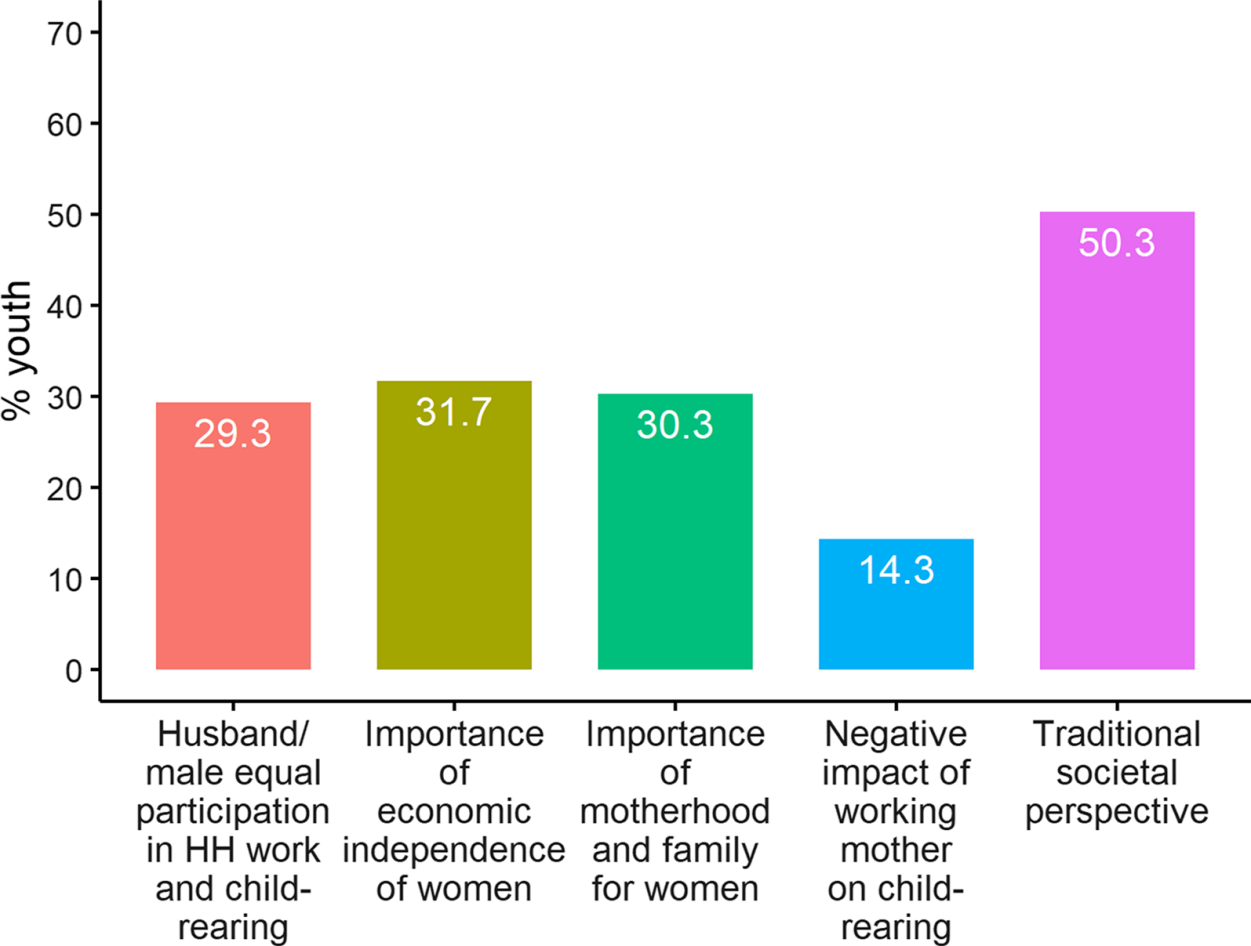
Percentage Emirati youths who agree (above median) with the gender equality statements, 2019

Around 14% of the youths agreed with statements suggesting a negative impact of a working mother on child-rearing (Fig. 1). Thirty-three percent of the youths agreed that motherhood and family are more important for women. Twenty-nine percent of the youths agreed on equal participation of husband/males in HH work and child-rearing. Fifty percent of the youths agreed with a traditional societal perspective on marriage, and 32% of the youths agreed with the importance of economic independence for women.

Male youths agreed more (male: 25%, female: 7%) with the negative impact of working mother on child-rearing and importance of motherhood and family for women (male: 39%, female: 24%) than female youths (Table 2). In contrast, males demonstrated less agreement on equal participation of husbands/males in HH work (23%) and women’s economic independence (44%) compared to females. Surprisingly, there was no gender differences among youths who agreed with a traditional societal perspective (males: 51%, females: 50%).

**Table 2:** Percentage Emirati youths who agree (above median) with the gender equality statements by background characteristics, 2019

	Negative impact of working mother on child-rearing	Importance of motherhood and family for women	Husband/male equal participation in HH work and child-rearing	Traditional societal perspective	Importance of economic independence of women
Gender					
Male	24.6*	38.9*	23.0*	50.8	27.0
Female	6.9	24.1	33.9	50.0	35.1
Marital status					
Unmarried	14.6	30.7	23.6	49.4	31.5
Married	11.5	26.9	37.7	51.6	32.0
Age					
19–22	16.3	32.0	29.2*	49.6	32.1
23–30	11.5	27.9	30.8	57.7	26.9
Father’s education					
Primary or less	10.2	26.5	28.6	63.3	30.6
Basic secondary	18.8	37.5	31.3	47.9	29.2
High School	14.9	26.6	26.6	47.9	29.8
Undergraduate	15.1	33.7	31.4	52.3	33.7
Graduate and above	10.0	20.0	20.0	25.0	35.0
Mother’s education					
Primary or less	15.2	30.4	28.3	54.3	30.4
Basic secondary	17.4	34.8	34.8	56.5	32.6
High School	11.9	27.5	24.8	49.5	31.2
Undergraduate	14.5	26.3	30.3	44.7	32.9
Graduate and above	26.7	40.0	33.3	40.0	20.0
Total	14.3	30.3	29.3	50.3	31.7

*Indicates that the Chi-square test for association was statistically significant with p-value ≤ 0.05

Unmarried youths compared to married youths were more in agreement with less gender equitable statements such as—negative impact of working mother on child-rearing, importance of family and motherhood for women and traditional societal perspective. Fathers’ education did not show any definitive pattern with gender equality statements except for the statement on traditional societal perspective. The percentage of youths agreeing with traditional societal perspective declined with increasing father’s education and similar pattern could be seen for mother’s education as well. Less males (27%) agreed with the importance of economic independence of women than females (35%). Higher proportion of younger youths of 19–22 years: 32% agree with the importance of economic independence of women than those aged 23–30 years: 27%.

A chi-square test of association showed that among the selected background characteristics only gender was significantly associated with the Negative impact of working mother on child-rearing, Importance of motherhood and family for women and husband/male equal participation in HH work and child-rearing. Age was significantly linked only with Husband/male equal participation in HH work and child-rearing.

**Fig. 2:**
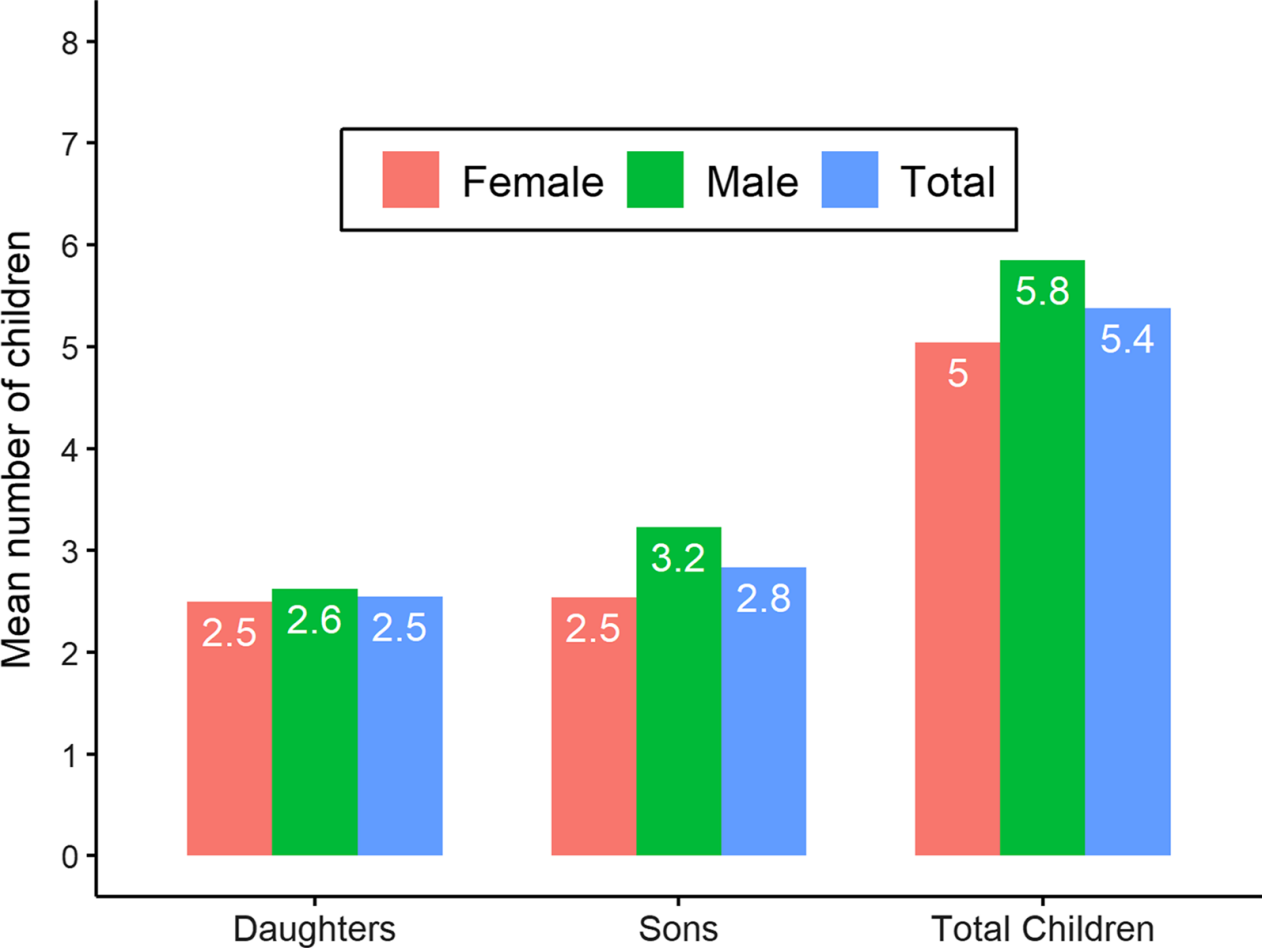
Average ideal number of children among Emirati youths by gender, 2019

Male youths mean ideal number of children is higher (5.8) than female youths (5.0) (Fig. 2). On average, desire for number of sons was higher than desire for number of daughters. Males, on average, indicated an ideal number of 3.2 sons and 2.6 daughters. Among females, the desired number of sons and daughters was almost equal.

Among youths who agreed with the negative impact of working mother on child-rearing average ideal number of children was higher (6.1) than those who disagreed with these statements (5.3) (Table 3). Similarly, among those who agreed on the importance of motherhood and family for women, average ideal number of children was 5.8 as compared to 5.2 among those who do not agree. Youths who did not agree with equal participation of husband/male in HH work and child-rearing wanted more children than those who support equal participation of husband/male in HH work and child-rearing.

**Table 3:** Average ideal number of children among Emirati youths by selected gender equality statements, 2019

	Ideal number of children	Ideal number of sons	Ideal number of daughters
Negative impact of working mother on child-rearing			
Disagree (Below median)	5.3*	2.8*	2.5
Agree (Above median)	6.1	3.3	2.8
Importance of motherhood and family for women			
Disagree (Below median)	5.2*	2.7	2.5
Agree (Above median)	5.8	3.1	2.7
Husband/male participation in HH work and child-rearing			
Disagree (Below median)	5.6*	2.9*	2.6*
Agree (Above median)	4.9	2.5	2.3
Traditional societal perspective			
Disagree (Below median)	5.5	2.9	2.6
Agree (Above median)	5.2	2.7	2.5
Importance of economic independence of women			
Disagree (Below median)	5.7*	3.0*	2.7*
Agree (Above median)	4.8	2.5	2.3
Average for total sample	5.4	2.8	2.6

*Indicates that the t-test for the equality of means was statistically significant with p-value < 0.05

Individuals who agreed with the importance of economic independence of women desired less children (5.7) than those who disagreed with it (4.8). Overall, ideal number of sons desired was higher than the ideal number of daughters among Emirati youths. T-test analysis showed that mean number of ideal children and mean number of sons were significantly different between those who agreed and disagreed with all gender equitable statements except for their perception on Traditional societal perspective. However, the difference in mean number of daughters was only significant for the perception on Husband/male participation in HH work and child-rearing.

The results showed that those who agreed with the equal participation of husband/males in HH work and child rearing IRR = 0.87) and importance of economic independence of women (IRR = 0.87) had statistically lower rate ratio for ideal number of children when all other characteristics were controlled (Table 4).

**Table 4:** Poisson regression results assessing relationship between gender equality statements and ideal number of children among Emirati youths, 2019

	IRR	95% CI	95% p-value
Negative impact of working mother on child-rearing			
Disagree			
Agree	1.03	0.90,1.17	0.66
Importance of motherhood and family for women			
Disagree			
Agree	1.07	0.96,1.19	0.24
Husband/male participation in HH work and child-rearing			
Disagree			
Agree	0.87*	0.79,0.97	0.01
Traditional societal perspective			
Disagree			
Agree	0.94	0.86,1.03	0.21
Importance of economic independence of women			
Disagree			
Agree	0.87*	0.78,0.97	0.01
Gender			
Male			
Female	0.84*	0.75,0.94	0.00
Age			
19–22			
23–30	1.06	0.95,1.18	0.28
Father’s education			
Primary or less			
Basic secondary	1.09	0.91,1.31	0.33
High School	1.04	0.85,1.26	0.73
Undergraduate	0.98	0.81,1.18	0.81
Graduate and above	0.97	0.78,1.21	0.79
Mother’s education			
Primary or less			
Basic secondary	1.00	0.83,1.22	0.97
High School	0.91	0.74,1.11	0.33
Undergraduate	0.93	0.75,1.14	0.47
Graduate and above	1.04	0.82,1.31	0.77
Time spent by father in HH activities	1.02	0.99,1.05	0.00
Time spent by mother in HH activities	1.00*	0.99,1.00	0.00
Social ladder	1.00	1.00,1.01	0.12
Total number of children born to mother	1.03*	1.01,1.05	0.00
Constant	4.13*	3.06,5.58	0.00

*p value ≤ 0.05

Females as compared to males appeared to have a rate 0.84 times lower for ideal number of children. Both mother’s and father’s education were not statistically associated with ideal number of children. Ideal number of children increased statistically with increase in time spent by mothers in HH work whereas it decreased with increase in time spent by fathers in HH work. Ideal number of children among youths increased with increase in number of children born to their mothers.

## Discussion

Present analysis revealed that though 50% of Emirati youths maintained a traditional perspective on marriage and 30% believed motherhood is most important for women, a small percentage of them supported husband/male participation in HH activities and child rearing, and economic independence of women. Alibeli et al. (2015) also supported changing gender perception towards increased equality within the country [53].

Youths attitude towards gender equality statements revealed a gender differential. Less males supported the idea of female economic independence and husband/male participation in HH activities, and more males agreed with negative impact of working mother and importance of motherhood for females. Similar gender differences in attitudes towards gender equality have been found in previous studies conducted in Middle East and North Africa (MENA) countries [53–55].

The difference between the desired number of sons and daughters was significant among those who agreed with negative impact of working mother on child-rearing and importance of motherhood. Among those who agreed with traditional perspective statements, the difference between desired number of sons and daughters was small. Overall, similar to other traditional societies, present study found Emirati youth desire more boys than girls [56, 57].

A parent’s role in household activities, gender and number of children born by mother were associated with the ideal number of children among Emirati youth. Thus, the hypothesis H2: that there is association between number of siblings in the parental family and housework distribution between father and mother and ideal number of children was confirmed. The more egalitarian the youths’ family (with greater involvement of father household activities) was the lower the desired number of children. Conversely, students who have more siblings with the same mother tend to have a higher ideal number of children. This results is in line with the previous literature that suggests that number of children born by mother or childhood family size has a positive effect on expected family size that remains stable when controlling for the socioeconomic status of the parents [51].

Studies in past have reported being a male was positively associated with higher fertility desire [48]. Our study outcomes report similar finding that young males desire higher number of children. Increased direct and indirect childbirth costs explain decline in fertility [15]. As women now have more opportunities outside home, the decision to have children is becoming increasingly costly in terms of foregone work [39, 58].

Only two out of the five domains of gender equality attitudes showed statistically significant association with the ideal number of children: the importance of economic independence of women and equal participation by husband/males in household activities and child rearing. Thus, our study concludes that despite an increase of education and employment opportunities for Emirati women in recent years, societal attitudes toward gender equality roles remain traditional. The disparity in household workload between men and women maybe a contributing factor to the lower desired number of children. Existing literature also supports that division of family work has some influence on fertility intentions [58, 59].

According to McDonald (2000a and 2000b), the gap between high gender equality in institutions such as education and market employment and lower level of equality in family life have contributed to low fertility in advanced countries [39, 43]. Working women need help and support from their partners in both HH work and child rearing, in absence of which they prefer having a smaller number of children. Students-participants of the study who declared more egalitarian attitudes (those who agreed that husbands should equally participate in the housework and childcare, and those who agree that female economic independence is important) would want less children if they perceive a disproportionate burden on women with housework responsibilities alongside expectations of professional success.

This study suggests that more young Emirati females are in favour of the gender equality statements than males creating an imbalance in gender equality between couples. While women with gender equality attitudes may have lower fertility rates [15], Torr and Short (2004) stated that if both partners (husband and wife) share the same world-views that couples are more likely to have an additional child than those who have different gender attitudes which creates imbalance in values [60].

The current study provides useful insights into the relationship between gender role perceptions and ideal number of children in the context of the UAE. However, there are few limitations. Firstly, the study sample was restricted to Emirati undergraduate student youth, which may not represent the entire youth population of the UAE. However, obtaining higher education is perceived as a must thing by the national families and a kind of mandatory for the school graduates. Further research with a more diverse sample would be a good tool for evidence-based decision making and comprehensive family policies in the UAE.

Secondly, the study primarily was focused on examining gender role perceptions and their association with the ideal number of children. While this provides valuable insights, other important factors such as economic factors, and cultural norms could not be extensively explored due to survey data limitations.

Furthermore, the study design was cross-sectional, which limits the ability to establish causal relationships between the predictors and ideal or factual number of children participant have or want to have.

## Conclusions

Almost all Arab countries, including the UAE, have been facing continuous decline in fertility rates. In fact, the UAE government has implemented strong family support policies for its citizens. Despite these efforts, the fertility rates in the UAE continue to decline.

This study highlights a significant gap between the ideal number of children desired by Emirati youths and the current fertility rates. This disparity indicates there is potential to improve country’s fertility rates. Furthermore, this study investigated the factors associated with the ideal number of children among Emirati youth to provide better understanding of the socio-economic and cultural factors that influence fertility drop in the UAE. The results of the study showed that young people who agreed with the importance of women’s economic independence and equal participation by husbands/males in household activities and childrearing have a smaller ideal number of children. This suggests that youth with more gender-equal perceptions want to have less children.

European demographic researchers argue that a positive influence of gender equality on women’s reproductive goals can be expected if the double burden of women is reduced [10]. Considering this and based on the study findings, it can be recommended that efforts to raise gender equality attitudes among the population and strengthening institutional support for childbearing should go hand in hand. This may be helpful to avoid falling into a long-term low fertility trap. Table 5

**Table 5:** The list of gender roles items collected via the survey and their sources

Survey item	Survey/questionnaire borrowed from	References
A working mother can establish just as warm and secure a relationship with her children as a mother who does not work	ISSP/GSS (2022) Study: ZA5900—International Social Survey Programme: Family and Changing Gender Roles V—ISSP 2022 A working mother can establish just as warm and secure a relationship with her children as a mother who does not work	https://issp.org/data-download/by-topic/
A pre-school child is likely to suffer if his or her mother works	Adjusted form WVS/EVS (2017/2022) Q28. When a mother works for pay, the children suffer (Pre-school child suffers with working mother)	Inglehart, R., C. Haerpfer, A. Moreno, C. Welzel, K. Kizilova, J. Diez-Medrano, M. Lagos, P. Norris, E. Ponarin & B. Puranen et al. (eds.). 2014. World Values Survey: All Rounds—Country-Pooled Datafile Version: https://www.worldvaluessurvey.org/WVSDocumentationWVL.jsp. Madrid: JD Systems Institute
A job is alright but what most women really want is a home and children	ISSP (2017/2022) Study: ZA5900—International Social Survey Programme: Family and Changing Gender Roles IV—ISSP 2012 V8—Q1d Working woman: What women really want is home and kids	https://search.gesis.org/variables/exploredata-ZA5900_VarV8
Having a job is the best way for a woman to be an independent person	EVS (2010/2020) Having a job is the best way for a woman to be an independent person	EVS (2020): European Values Study Longitudinal Data File 1981–2008 (EVS 1981–2008). GESIS Data Archive, Cologne. ZA4804 Data file Version 3.1.0, https://europeanvaluesstudy.eu/methodology-data-documentation/evs-methodology/
In general, fathers are as well suited to look after their children as mothers	PEW/National Parent Survey (2013–2022)	Pew Research Center for the People & the Press. Roper Center at Cornell University. https://www.pewresearch.org/question-search/
Men should take as much responsibility as women for the home and children	Gallup (2017/2022) Statement Adjusted from Gender roles surveys	https://news.gallup.com/poll/283979/women-handle-main-household-tasks.aspx
When jobs are scarce, men have more right to a job than women	WVS/EVS (2017/2022) Q33 When jobs are scarce, men should have more right to a job than women	Inglehart, R., C. Haerpfer, A. Moreno, C. Welzel, K. Kizilova, J. Diez-Medrano, M. Lagos, P. Norris, E. Ponarin & B. Puranen et al. (eds.). 2014. World Values Survey: All Rounds—Country-Pooled Datafile Version: https://www.worldvaluessurvey.org/WVSDocumentationWVL.jsp. Madrid: JD Systems Institute
Being a housewife is just as fulfilling as working for pay	WVS/EVS (2017/2022) Q32 Being a housewife is just as fulfilling as working for pay	Inglehart, R., C. Haerpfer, A. Moreno, C. Welzel, K. Kizilova, J. Diez-Medrano, M. Lagos, P. Norris, E. Ponarin & B. Puranen et al. (eds.). 2014. World Values Survey: All Rounds—Country-Pooled Datafile Version: https://www.worldvaluessurvey.org/WVSDocumentationWVL.jsp. Madrid: JD Systems Institute
It is better for a family if husband earns more than wife	Adjusted from PEW (2013) Do you agree or disagree with the following statement? It’s generally better for a marriage if the husband earns more money than his wife	Pew Research Center for the People & the Press. Roper Center at Cornell University. https://www.pewresearch.org/question-search/
When deciding about the marriage partner the most important is to follow the advice of parents	Designed by authors	
Both husband and wife should equally do the work on household (cooking, cleaning, shopping, repairing etc.)	Designed by authors	

## Data Availability

The dataset generated and analysed during the current study is not publicly available, but it is available from the corresponding author upon request.

## Annex

See Table 5.

## Abbreviations

UAE: United Arab Emirate
GCC: Gulf Cooperation Council
UN: United nations
GII: Gender inequality index
MENA: Middle East and North Africa

## Footnotes

1 GII, is a measure of gender inequalities in three important aspects of human development— health, empowerment, and economic status, sheds light on the position of women in 162 countries (Human Development Report 2019).

2 All the analysis is done in the software STATA-16.
